# 67 kDa laminin receptor (67LR) in normal and neoplastic hematopoietic cells: is its targeting a feasible approach?

**Published:** 2016-11-01

**Authors:** Nunzia Montuori, Ada Pesapane, Valentina Giudice, Bianca Serio, Francesca W Rossi, Amato De Paulis, Carmine Selleri

**Affiliations:** 1 Department of Translational Medical Sciences, University of Naples Federico II, Naples, Italy; 2 Department of Medicine and Surgery, University of Salerno, Salerno, Italy

**Keywords:** lamininreceptor, laminin, hematopoietic stem cells, leukemia, multiple myeloma

## Abstract

The 67 kDa laminin receptor (67LR) is a non-integrin cell surface receptor for laminin (LM) that derives from a 37 kDa precursor (37LRP). 67LR expression is increased in neoplastic cells and correlates with an enhanced invasive and metastatic potentialin many human solid tumors, recommending this receptor as a new promising target for cancer therapy. This is supported by in vivo studies showing that 67LR downregulation reduces tumour cell proliferation and tumour formation by inducing apoptosis. 67LR association with the anti-apoptotic protein PED/PEA-15 activates a signal transduction pathway, leading to cell proliferation and resistance to apoptosis.

However, the main function of 67LR is to enhance tumor cell adhesion to the LM of basement membranes and cell migration, two crucial events in the metastasis cascade. Thus, inhibition of 67LR binding to LM has been proved to be a feasible approach to block metastatic cancer cell spread.

Despite accumulating evidences on 67LR overexpression in hematologic malignancies, 67LR role in these diseases has not been clearly defined. Here, we review 67LR expression and function in normal and malignant hematopoietic cells, 67LR role and prognostic impact in hematological malignancies and first attempts in targeting its activity.

## I.INTRODUCTION

The 67kDa laminin receptor (67LR) is a non integrin cell surface receptor for extracellular matrix (ECM) able to bind with high affinity laminin-1 (LM), the major glycoprotein of basement membranes [1]. The primary function of 67LR is to promote tumor cell adhesion and migration to LM, crucial steps in tissue invasion and metastasis, by binding LM with high affinity (Kd=2×10^−9^ M). cDNA clones coding for human and mouse 67LR encode a protein with a molecular weight of 32kDa and an apparent electrophoretic mobility of about 37kDa. This polypeptide was identified as the precursor of 67LR, and named 37kDa laminin receptor precursor (37LRP) [2].

The mechanism by which 37LRP is converted into the mature 67LR is still unclear [3,4]. 37LRP is abundantly localized in the cytoplasm, where it acts as a multifunctional protein involved in the translational machinery and in ribosome assembly, and in the nucleus, tightly associated with nuclear structures [5].67LR, the mature form of the receptor, is localized in the cell membrane, from which it is internalized via early-endosomes and lysosomal-mediated degradation [6]. On the cell surface, 67LR is able to interact with α6β4 integrin; both receptors are co-expressed and physically associated in a complex that recognize different sites on LM, increasing the affinity of the binding [7]. Upon binding 67LR, LM interacts more efficiently with integrins and becomes more sensitive to proteolytic enzymes, releasing fragments endowed with chemotactic activity [8]. Three regions of 37LRP/67LR are involved in LM binding: (i) repeated sequences (TWEDS) at the C-terminal, (ii) a direct laminin binding region (aminoacids 205–229) [9] and (iii) a heparan sulfate dependent LM binding region (amino acids 161–180), also called peptide G, and containing the palindromic sequence LMWWML, responsible for LM binding [10].

All the three LM-binding sites of the receptor bind the same minimal YIGSR region of the β1 chain of LM [11]. As a membrane receptor, 37LRP/67LR is also a receptor for viruses, bacteria and prions [5,12].67LR is overexpressed in neoplastic cells and correlates with an enhanced invasive and metastatic potential in many solid tumors [13–17]. 67LR role in metastatic diffusion is well documented and mostly rely in its ability to mediate adhesion to the LM of basement membranes of epithelia and endothelia and to mediate trans-endothelial migration of metastatic cancer cells [18]. 67LR role in hematological malignancies has not been clearly defined, even though many reports have been produced on its expression, function and inhibition on normal and malignant hematopoietic cells.

## II. 37LRP/67LR FUNCTION AND TARGETING IN SOLID TUMORS

Given its importance in solid tumor progression, 67LR represents a suitable target for cancer therapy and different approaches have been used to inhibit its function, in order to contribute to metastasis prevention and/or treatment (reviewed in 19).Different strategies used against this receptor were able to reduce the invasive potential of HT1080 fibrosarcoma cells [20]. 37LRP/67LR is able to affect tumor progression also by promoting angiogenesis; indeed, a receptor specific antibody inhibited endothelial tube formation [21].37LRP/67LR is involved in the maintenance of cellular viability and reduction of its expression induced apoptosis of cancer cells [22]. The role of 37LRP/67LR in apoptosis was also confirmed by the finding by our group of a structural and functional association between 67LR and the anti-apoptotic protein PED/PEA-15 [23].

A recent study reveals a novel function of 37LRP/67LR: siRNA treatment of 37LRP/67LR resulted in a significant decrease of telomerase activity [24].Recently, our group searched, by a virtual screening (VS) approach, small molecules able to specifically target 67LR. VS is a computational method that allows the identification of new therapeutics for a specific biological target from large chemical libraries [25]. This study led to the identification of a specific inhibitor of 37LRP/67LR, NSC47924. This compound specifically inhibited cell adhesion and migration to LM, as well as cell invasion. A subsequent hierarchical similarity search with NSC47924 allowed the refinement of this lead compound, identifying additional four compounds able to inhibit cell binding to LM and to block *in vitro* cancer cell invasion, exhibiting a lower Ic_50_ as compared to NSC47924 [26].

These small molecules are cell-permeable and orally available, the most important advantage in respect to monoclonal antibodies. Moreover, they showed a short half-life, high specificity and low toxicity, thus may be of considerable clinical benefit in tailoring personalized target therapies in cancer.

## III. 37LRP/67LR EXPRESSION AND FUNCTION IN NORMAL HEMATOPOIETIC STEM CELLS

HSCs normally resides within the bone marrow (BM) and can be mobilized into the circulation by chemotherapy or cytokine treatment [27]. The most common mobilizer is the granulocyte-colony stimulating factor (G-CSF). GCSF-mobilized HSCs are increasingly used in stem cell transplantation (SCT) for the relative ease of collection, the higher yield and the shorter time to engraftment than BM stem cells [28].

We have demonstrated that 67LR expression is increased in G-CSF-mobilized HSCs, as compared with BM HSCs, and significantly correlated with mobilization efficiency [29]. During G-CSF–induced HSC mobilization, the expression of laminin receptors switched from α6 integrins, which mediated LM-dependent adhesion of steady-state human BM HSCs, to 67LR, responsible for G-CSF–mobilized HSC migration toward LM. This switch in the expression of LM receptors also induced a change in the signal transduction pathway activated in response to LM binding. In vitro G-CSF treatment, alone or combined with exposure to marrow-derived endothelial cells, induced 67LR up-regulation in marrow HSCs; moreover, anti-67LR antibodies significantly inhibited transendothelial migration of G-CSF–stimulated marrow HSCs. Finally, G-CSF–induced mobilization in mice was associated with 67LR up-regulation both in circulating and marrow HSCs, and anti-67LR antibodies significantly reduced HSC mobilization.

Engraftment of HSCs to BM after transplantation is a key factor in SCT. G-CSF-induced 67LR overexpression on G-CSF mobilized HSCs, could play a crucial role also in HSC homing back to BM by mediating interactions with the basement membranes of vascular endothelia and subsequent cell migration. Indeed, 67LR also promoted homing to BM of transplanted HSCs, playing a key role in erythroid progenitor and precursor cells lodgment within the BM [30]. Thus, 67LR overexpression occurs in BM and circulating normal HSCs after cytokine stimulation and regulates HSC trafficking from and to BM. These findings further support a model in which HSC mobilization could represent a physiologic counterpart of leukemic and metastatic cell spread.

## IV. 37LRP/67LR EXPRESSION AND TARGETING IN CHRONIC LYMPHATIC LEUKEMIA

B-cell chronic lymphocytic leukemia (CLL) is a heterogeneous group of diseases with various B-cell membrane markers expression and clinical course [31]. Despite the identification of genetic and phenotypic markers that correlate with prognosis, the biological basis of this clinical variability remains unclear [32].

In CLL, 37LRP/67LR is widely expressed and 37LRP is considered as an oncofetal antigen (OFA), thus often referred to as OFA/iLRP (oncofetal antigen/immature laminin receptor) [33]. Oncofetal antigens are conserved tumor-associated antigens or transplantation antigens expressed on the surface of human tumors and on fetal cells but not on normal adult tissues. OFAs are able to induce an immune response against tumors as well as a tolerogenic response, linked to cancer progression [34]. Dendritic cells (DCs) primed with OFA/iLRP or transfected with RNAs specific to OFA/iLRP induced a T-cell immune response against hematological malignancies, in particular acute myeloid leukemia (AML) and chronic lymphatic leukemia (CLL) cells. In a murine B-cell lymphoma model, treatment with syngeneic DCs transfected with OFA/iLRP-coding RNA resulted in powerful antitumor effect [35]. There is also evidence of a humoral response against OFA/iLRP: pre-existing antibodies (Abs) to OFA/iLRP have been detected in sera of CLL patients. Patient Abs to OFA/iLRP were cytotoxic in vitro and individuals with an anti-OFA/iLRP humoral response had a more favorable prognosis. OFA/iLR Abs were cytotoxic and exerted also a role in the graft-vs-leukemia effect in CLL [36].

Confirming its nature of immune stimulating tumor associated antigen, high expression of the protein OFA/iLR correlated with mutated IGVH status and predicted for a favorable prognosis in CLL [37]. These results are in agreement with reports on the ability of anti -37LRP/67LR monoclonal antibodies (MoAbs) to block neoplastic B cell proliferation in vitro and in vivo. Two MoAbs, BV-15 and BV-27, showed anti-metastatic activity in the A20 B-cell leukemia model. Only BV-27 was growth-suppressive in vitro; however, both antibodies suppressed A20 cell attachment to LM [38]. Thus, inhibition of LM attachment seems crucial for the inhibitory effect, as reported with antibodies targeting both the immature and mature forms of the receptor or with treatments that down-regulate the expression of 37LRP/67LR in solid tumors [19,39]. These MoAbs could be used therapeutically even though it is not clear whether they exert their action by effector functions (Ab or complement dependent cytotoxicity) or by their action (cell growth inhibition and/or blocking of cell binding to LM).

Epigallocatechin-3-gallate (EGCG) is the major polyphenol of green tea; it is a small molecule that functions as an antitumor and antiangiogenic agent. EGCG induces cell death and cell cycle arrest and 67LR was identified as a receptor able to mediate its anti-cancer activity [40]. In contrast with previous results showing that 67LR inhibition, through MoAbs, blocked neoplastic B lymphocyte proliferation, a phase II clinical trial demonstrated that 67LR stimulation by oral Polyphenon^ETM^ was well tolerated and 29 of 43 CLL patients (67%) showed evidence of a biological response with decreased lymphadenopathy and/or absolute lymphocyte count [41]. Moreover, there was a significant correlation between EGCG susceptibility and 67LR expression in CLL cells and Vardenafil, a clinically available phosphodiesterase inhibitor, potentiated the killing effect of EGCG on CLL cells [42]. The molecular mechanism of Vardenafil action on EGCG-induced 67LR stimulation and tumor cell killing was better elucidated in multiple myeloma cells (see below).

## V. 37LRP/67LR EXPRESSION AND FUNCTION IN MULTIPLE MYELOMA

Multiple myeloma (MM) represents a B cell malignancy, characterized by a monoclonal proliferation of malignant plasma cells. During disease evolution, terminally differentiated B cells preferentially accumulate in the BM. LN stimulated *in vitro* migration of human and murine MM cells, through its binding to 67LR, overexpressed on MM cells. 67LR inhibition by the LM-derived peptide CDPGYIGSR, resulted in a decreased homing of MM cells to the BM in a murine in vivo model [43]. Thus, LM acts as a chemoattractant for MM cells by interaction with 67LR and this interaction might be important during the trafficking of MM cells, as already demonstrated for normal HSCs [29,30]. 67LR is also involved in lymphoma cells homing to lymph nodes and in their trafficking to specific organs [44].

In MM, EGCG was able to induce inhibition of cell growth and apoptosis *in vitro* and *in vivo*. Silencing of 67LR resulted in abrogation of EGCG-induced apoptosis, confirming the role of 67LR in EGCG-mediated growth inhibition in MM cells [45].EGCG induced apoptosis through 67LR be determining phosphorylation of PKCδ and activation of acid sphingomyelinase (aSMase). cGMP is a critical mediator of 67LR-dependent PKCδ/aSMase activation and MM apoptosis. EGCG induces nitric oxide (NO) production through 67LR-dependent activation of Akt and endothelial nitric oxide synthase (eNOS). NO increases the intracellular level of cGMP, that induces apoptosis by activating PKCδ/aSMase pathway. aSMAse acts on sphingomyelin [46] to generate ceramide, which induced lipid-rafts clustering, critical for apoptosis. Orally administered EGCG activated PKCδ and aSMase in a murine MM xenograft model [47].In MM cells, phosphodiesterase 5 (PDE5), a major negative regulator of cGMP, is overexpressed and is able to reduce 67LR-mediated apoptosis induced by EGCG. Thus, Vardenafil, a PDE5 inhibitor, induced an enhancement of the EGCG-activated 67LR-dependent apoptosis, through amplification of the downstream effectors PKCδ and aSMase, and prolongation of the survival time in a mouse xenograft model [46,47].

## VI. 37LRP/67LR EXPRESSION AND FUNCTION IN ACUTE MYELOID LEUKEMIA

Acute myeloid leukaemia (AML) is an aggressive blood cancer caused by the proliferation of immature myeloid cells. The genetic abnormalities underlying AML affect signal transduction pathways, transcription factors and epigenetic modifiers. The genetic landscape of AML cells could exert a direct effect on the anti-leukemic immune responses [48]. Thus, 67LR expression and function in AML could play a critical role in the evolution and prognosis of the disease.

We detected enhanced 67LR expression in 40% of 53 de novo AMLs, which frequently exhibited monocytic or myelomonocytic morphology. We did not detect 67LR expression in normal BM hematopoietic cells, in precursor-B acute lymphoblastic leukemia, in chronic lymphocytic leukemia, or in chronic myeloid leukemia in chronic phase. 67LR overexpression corresponded to a higher adhesion to LM. In contrast with 67LR behavior in solid tumors, no statistically significant difference was found between 67LR expression and any hematological characteristic of the disease at diagnosis, nor between 67LR expression and outcome of the disease as measured by complete remission rate, disease-free survival, or overall survival [49].

A more recent study demonstrated 67LR expression influenced the characteristics of AML cells toward an aggressive phenotype and increased the expression of GM-CSF receptor. Indeed, increased expression of 67LR was significantly related to elevation of white blood cell count, lactate dehydrogenase, and poorer survival among AML patients. Forced expression of 37LRP/67LR enhanced proliferation, cell-cycle progression, and antiapoptosis of AML cells associated with phosphorylation of STAT5, in the absence of stimulation LM. There was a significant relationship between the expression of 67LR and GM-CSFR in acute myeloid leukemia samples, with enhanced GM-CSFR signaling [50].

This observation is not surprising; indeed, a previous work showed that 67LR was an interacting protein of both the alpha and beta subunits of GM-CSFR. Whereas GM-CSF functions by engaging the alpha and beta subunits into receptor complexes, 67LR inhibited GM-CSF-induced receptor complex formation. 67LR engagement by LM relieved the LR inhibition of GM-CSFR. These findings provided a mechanistic basis for enhancing host defense cell responsiveness to GM-CSF at transendothelial migration sites, where 67LR is engaged by LM, while suppressing it in circulation [51].

EGCG-induced cell death through cGMP/aSMase axis activation and lipid raft clustering was described also in AML [52] and in chronic myeloid leukemia (CML) [53].

## VII. CONCLUSIONS

Mobilization of HSCs into the blood following treatment with chemotherapy or cytokines mimics the enhancement of the physiologic stem-cell release in response to stress and inflammatory signals and results from changes in the adhesion profile of HSCs, facilitating their egress from BM. Cytokine-stimulated HSCs, leukemia and multiple myeloma cells, as metastatic cells from solid tumors, activate a 67LR-derived signaling pathway, which leads to cell dissemination and trafficking through the host.

36LRP/67LR is overexpressed in CLL and AML, but with a different prognostic impact. 37LRP/67LR overexpression in CLL has a positive impact, due the stimulation of an anti-leukemia effect. The same effect is not observed in AMLs in which 67LR upregulation is correlated, as in solid tumors, to increased aggressiveness and poorer response to treatments.

Biological agents inhibiting 67LR binding to LM, such as antibodies and peptides, could represent an efficient tool to target CLL and myeloma cells and the activity of EGCG in CLL has been already proved by an early clinical trial. Concurrently, many reports indicate that EGCG activity through 67LR may help in the design of new strategies to also treat AML and MM.

It is intriguing that a 67LR stimulating small molecule, such as EGCG, can exert the same antitumor effect of 67LR inhibitory molecules. Most probably, clarification of the molecular mechanism of action of new small molecules inhibiting LM binding to 67LR, such as NSC47924 and its analogs, will help to clarify this issue.

## Figures and Tables

**Figure 1:**
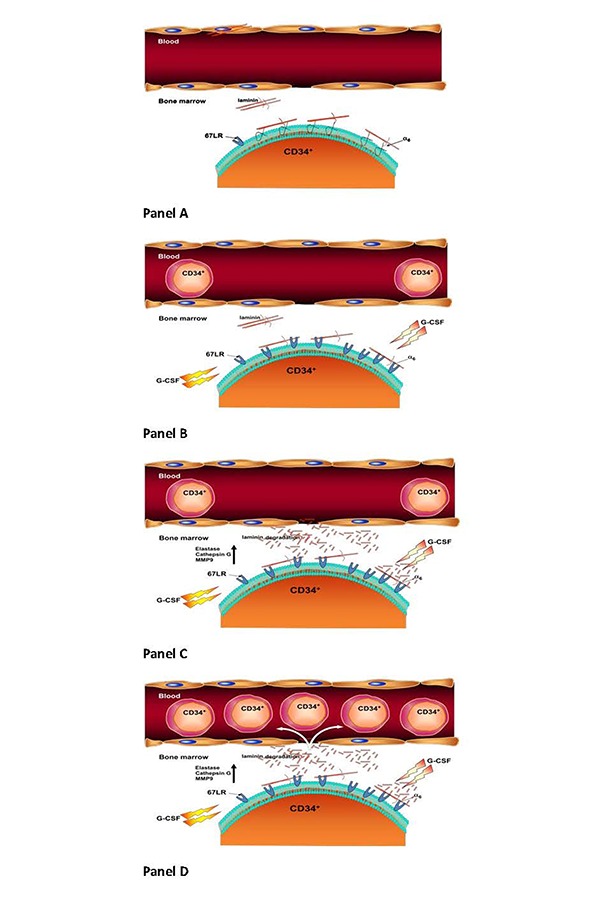
67LR involvement in hematopoietic stem cell (HSC) mobilization. **Panel A.** Steady-state interactions between HSCs and LM of the BM microenvironment are mediated by α6 containing integrin receptors transducing proliferative signals. **Panel B.** G-SCF modification of HSC ECM receptor profile leads to α6 integrin downregulation and 67LR overexpression. **Panel C.** Overexpressed 67LR, through LM binding, transduces motility signals, stimulates the secretion of proteolytic enzymes, modifies LM structure with the release of motility fragments. **Panel D.** 67LR engagement by LM enhances HSC release from the BM; mobilized HSCs show increased 67LR expression important for their subsequent homing to BM.
